# Periodontitis-associated salivary microbiota exacerbates systemic osteoclastogenesis via gut modulation and tryptophan metabolism suppression in ovariectomized mice

**DOI:** 10.1038/s41368-025-00415-2

**Published:** 2026-01-27

**Authors:** Nannan Wang, Jun Qian, Min Wang, Lili Li, Wenzheng Liao, Rixin Chen, Hua Nie, Ruiyang Ge, Fangfang Sun, Fuhua Yan

**Affiliations:** 1 Nanjing Stomatological Hospital, Affiliated Hospital of Medical School, Institute of Stomatology, Nanjing University, Nanjing, China; 2 Shenzhen Hospital, Southern Medical University, Shenzhen, China

**Keywords:** Periodontitis, Oral diseases

## Abstract

Epidemiological studies have highlighted an association between periodontitis and osteoporosis. However, the mechanism underlining this association remains unclear. Here, we revealed significant differences in the salivary microbiota between periodontally healthy individuals and periodontitis patients, with periodontitis patients exhibiting increased salivary microbiota diversity and an elevated abundance of pathogenic bacteria. Using an ovariectomized (OVX) mouse model, we demonstrated that the salivary microbiota from periodontitis patients exacerbated bone destruction by modulating the gut microbiota. Metabolomic analysis revealed that the periodontitis-associated salivary microbiota suppressed tryptophan metabolism. The tryptophan metabolite indole-3-lactic acid (ILA) directly inhibited osteoclast formation and differentiation. In OVX mice treated with periodontitis salivary microbiota, supplementation with ILA effectively suppressed osteoclastogenesis and alleviated the detrimental effects of periodontitis-associated salivary microbiota on systemic bones. In summary, our data demonstrate that periodontitis can affect systemic bone metabolism via the oral–gut axis and that ILA supplementation serves as a potential therapeutic option to mitigate these adverse effects.

## Introduction

Periodontitis is a chronic inflammatory disease of the periodontal supporting tissues initiated by dental plaque microorganisms. The Global Burden of Disease Study revealed a striking 91.5% increase in severe periodontitis cases between 1990 and 2021, with the global prevalence reaching 12.5% in 2021.^1^ Beyond its local manifestations of tooth mobility and loss, this oral condition is significantly associated with systemic pathologies, including cardiovascular diseases, malignancies, diabetes mellitus, rheumatoid arthritis, and osteoporosis.^2–6^ Osteoporosis, characterized by decreased bone mineral density (BMD) and microarchitectural deterioration, presents a substantial public health burden with a global prevalence of 19.7% for osteoporosis and 40.4% for osteopenia.^7^ Emerging epidemiological evidence suggests a bidirectional relationship between these conditions: periodontitis patients exhibit elevated osteoporosis risk,^8,9^ while osteoporotic individuals with severe periodontitis have significantly reduced BMD levels.^9^ Although shared risk factors (aging, vitamin D deficiency, and smoking) partially explain this association,^10–15^ the precise mechanistic interplay remains elusive.

Recent advances highlight the gut microbiota as a pivotal modulator of bone metabolism through multiple pathways: microbial metabolite production, immune system regulation, and endocrine modulation.^16–18^ Our previous work established that periodontitis exacerbates bone homeostasis disruption in *ApoE*^*−/−*^ mice via alterations in the gut microbiota,^19^ suggesting the involvement of novel mechanisms involving the oral–gut–bone axis. Gut microbiota dysbiosis is intricately connected to oral microbial population shifts and intestinal colonization by orally derived pathogens.^20^ In periodontitis patients, the salivary microbiome undergoes significant enrichment of pathogenic organisms, with oral pathobionts such as *Porphyromonas gingivalis* and *Fusobacterium nucleatum* demonstrating the ability to translocate to the gastrointestinal tract, thereby compromising the gut’s microbial defense mechanisms.^21^ Our prior experimental work established that the administration of periodontitis-associated salivary microbiota into ovariectomized (OVX) animals by oral gavage markedly accelerates systemic bone loss,^22^ although the pathways driving this phenomenon remain undefined.

**Scheme 1:**
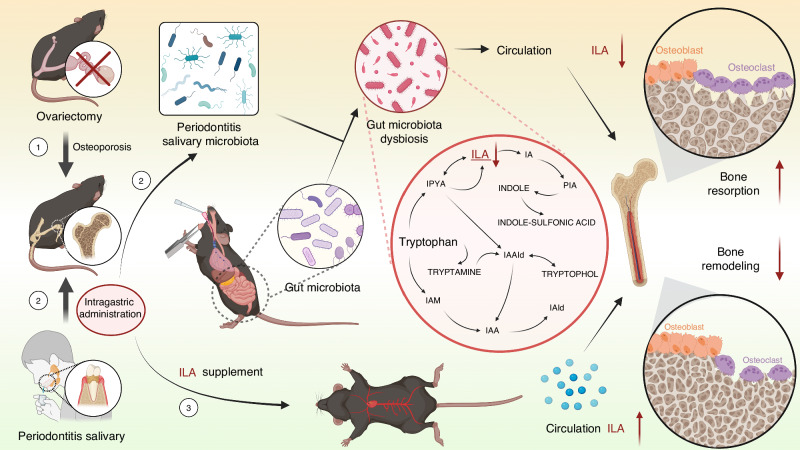
Schematic illustration of the experimental design and mechanism

The present study demonstrates significant differences in both the diversity and composition of the salivary microbiota between periodontitis patients and healthy individuals. Furthermore, we show that the salivary microbiota from periodontitis patients can induce bone loss in OVX mice. Using fecal microbiota transplantation (FMT), metabolomic analysis, and functional approaches, we investigated the mechanistic role of the gut microbiota and tryptophan metabolism in mediating periodontitis-induced bone resorption. Our findings specifically highlight indole-3-lactic acid (ILA). Supplementation with ILA effectively inhibited osteoclastogenesis and attenuated the detrimental skeletal effects induced by periodontitis-associated salivary microbiota in OVX mice. This work systematically elucidates the oral–gut–bone axis mechanisms through which periodontitis exacerbates bone metabolism in OVX mice, and proposes a potential therapeutic strategy for mitigating these adverse effects (Scheme 1).

## Results

### Periodontitis induces salivary microbiota alterations

Thirty-eight participants were enrolled, comprising 17 healthy donors (H group) and 21 patients with periodontitis (P group). The clinical characteristics of the participants are provided in Supplementary Table S1. The mean age of the P group ((43.95 ± 10.38) years) was significantly greater than that of the H group ((30.35 ± 4.37) years; *P* < 0.000 1). No significant difference was found between the sex ratios of the two groups.

**Fig. 1:**
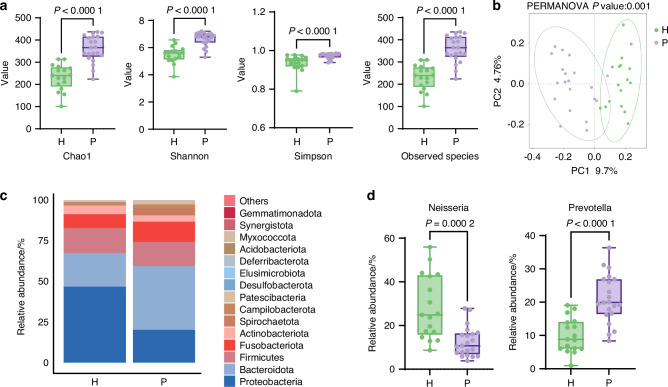
Periodontitis alters the composition of the salivary microbiota. **a** Alpha diversity indices (Chao1, Shannon, Simpson, and observed species) of the salivary microbiota based on 16S rRNA sequencing in healthy donors (H group, *n* = 17) and periodontitis patients (P group, *n* = 21). Box plots depict the median, interquartile range, minimum, and maximum values. **b** Principal coordinate analysis (PCoA) of the microbial communities in the H and P groups, based on the binary Jaccard distance. **c** Relative abundance of salivary microbes at the phylum level. **d** Comparation of the relative abundance of the dominant bacterial genera in the H and P groups. Box plots depict the median, interquartile range, minimum, and maximum values. *P* values were determined by the Mann‒Whitney U test (**a**, **d**) and PERMANOVA using the binary Jaccard distance (**b**). All the statistical tests were two-sided

To investigate the impact of periodontitis on the salivary microbiota, we performed 16S rRNA sequencing analysis between the P group and the H group. Alpha diversity analysis revealed significantly greater microbial richness in the P group than in the H group, as indicated by elevated Chao1 indices, along with increased Shannon and Simpson diversity indices and greater observed species counts (Fig. 1a). To further eliminate the influence of age, we adjusted for age differences between the two groups using regression analysis. The results revealed that the alpha diversity still differed significantly between the groups (*P* < 0.001), suggesting that periodontitis serves as an independent factor influencing the composition of the salivary microbiota. Principal coordinate analysis (PCoA) revealed distinct clustering patterns between groups, demonstrating that microbial communities were significantly restructured in association with periodontal disease (Fig. 1b). Notably, sex-specific variation did not significantly affect the beta diversity patterns in either cohort (Supplementary Fig. 1a, b). At the phylum level, the P group exhibited marked enrichment of *Bacteroidetes* (39.3% vs. 20.4%), *Fusobacteria* (12.5% vs. 8.5%), and *Spirochaetes* (4.6% vs. 0.2%), along with significant depletion of *Proteobacteria* (20.3% vs. 46.9%) and a reduced *Firmicutes*/*Bacteroidetes* ratio relative to controls (0.41 vs. 0.85; *P* < 0.000 1) (Fig. 1c). Genus-level analysis revealed a pronounced shift in microbial dominance: *Prevotella* predominated in the P group, whereas *Neisseria* prevailed in the H group (Fig. 1d). Linear discriminant analysis effect size (LEfSe) revealed significant enrichment of periodontal pathogens in the P group, particularly *Porphyromonadaceae* and *Prevotellaceae*. Conversely, the H group showed characteristic enrichment of commensal *Proteobacteria* and *Lactobacillales* (Supplementary Fig. 1c). Collectively, these findings demonstrate that periodontitis induces significant ecological alterations in the salivary microbiota, characterized by pathogen enrichment and disruption of commensal microbial networks.

### Salivary microbiota from periodontitis patients drives bone loss in OVX mice

**Fig. 2:**
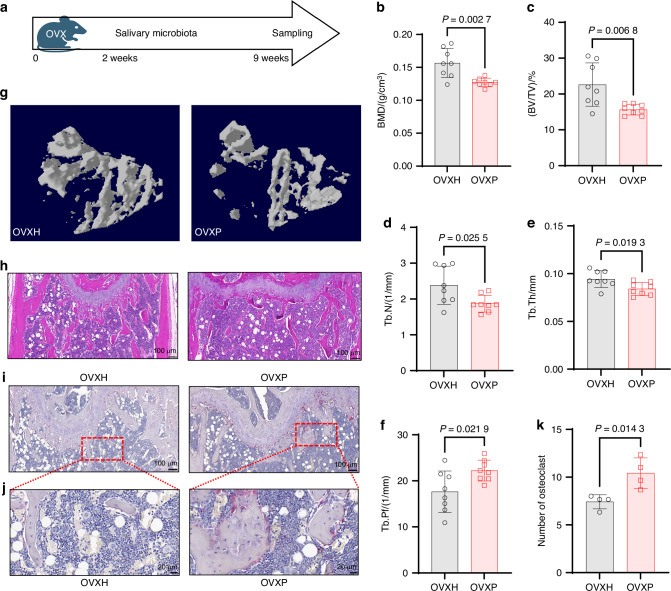
Salivary microbiota from periodontitis patients promotes bone loss and enhances osteoclastogenesis in OVX mice. **a** Schematic overview of the experimental design. Trabecular bone microarchitecture parameters of tibias from OVX mice treated orally with salivary microbiota from periodontitis patients (OVXP group, n = 8) or healthy donors (OVXH group, n = 8) for 7 weeks: **b** bone mineral density (BMD), **c** bone volume fraction (BV/TV), **d** trabecular number (Tb.N), **e** trabecular thickness (Tb.Th), and **f** trabecular pattern factor (Tb.Pf). **g** Representative 3D reconstruction of tibial trabecular bone (corresponding to **b**–**f**). Representative distal femoral sections stained with **h** H&E and **i** TRAP (scale bar: 100 μm) and **j** a magnified view of the region boxed in (**i**) (scale bar: 20 μm). **k** Quantification of TRAP-positive osteoclasts (*n* = 4 per group). The data are presented as the mean ± SD. *P* values were determined by Student’s *t*-test. All the statistical tests were two-sided

To investigate the impact of the salivary microbiota on osteoporosis, we established an animal model using ovariectomy. Two weeks after surgery, OVX mice received oral gavage of salivary microbiota from either healthy donors (OVXH group) or periodontitis patients (OVXP group) (Fig. 2a). Micro-CT analysis revealed significantly lower BMD in the OVXP group than in the OVXH group (Fig. 2b). Trabecular bone analysis of the proximal tibia revealed further microstructural deterioration in OVXP mice, characterized by significant decreases in bone volume fraction (BV/TV), trabecular number (Tb.N), and trabecular thickness (Tb.Th), as well as an increase in trabecular pattern factor (Tb.Pf) (Fig. 2c–g). Histologically, H&E staining revealed sparse, fragmented trabeculae with surface irregularities and a narrowed morphology in the OVXP group (Fig. 2h). Notably, increased numbers of TRAP-positive osteoclasts were observed within both the trabecular bone and the growth plate regions of OVXP mice (Fig. 2i–k). These findings indicate that the salivary microbiota of periodontitis patients enhances osteoclastogenesis and exacerbates osteoporosis in OVX mice.

### Gut dysbiosis driven by periodontitis exacerbates bone loss in OVX mice

**Fig. 3:**
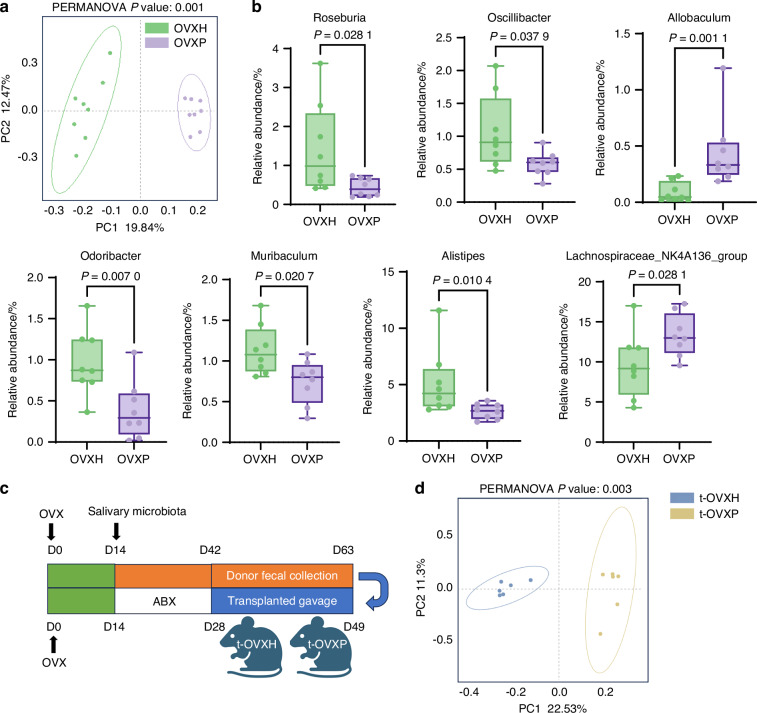
Salivary microbiota alters the composition of the gut microbiota. **a** Principal coordinate analysis (PCoA) of the gut microbiota in the OVXH and OVXP groups (n = 8 per group), based on the binary Jaccard distance. **b** Relative abundance of genera showing significant differences between the OVXH and OVXP groups, identified by random forest analysis. Box plots depict the median, interquartile range, minimum, and maximum. **c** Schematic of the fecal microbiota transplantation (FMT) experimental design. **d** Microbial beta diversity in recipient mice (t-OVXH and t-OVXP groups, n = 6 per group) assessed by PCoA. *P* values were determined by the Mann‒Whitney test (**b**) and PERMANOVA using the binary Jaccard distance (**a**, **d**). All the statistical tests were two-sided

Mounting evidence highlights the gut–bone axis as a pivotal pathway in metabolic bone disorders. To investigate the tripartite interplay among periodontal dysbiosis, gut microbiota, and bone metabolism in OVX mice, we used 16S rRNA sequencing to profile the gut bacteria of the OVXH and OVXP groups. Intriguingly, while the salivary microbiota of patients with periodontitis exhibited increased alpha diversity, no significant differences in the alpha diversity of the gut microbiota were observed between the OVXH and OVXP groups (Supplementary Fig. 2a). PCoA demonstrated distinct beta diversity clustering, indicating significant restructuring of the gut microbiota (Fig. 3a). LEfSe analysis identified significant enrichment of *Lachnospiraceae_NK4A136*, *Desulfovibrionaceae Erysipelotrichaceae*, *Paludicola*, and *Allobaculum* in the OVXP group, whereas *Alistipes*, *Rikenellaceae*, *Roseburia*, *Marinifilaceae*, *Odoribacter*, *Oscillibacter*, and *Muribaculum* were the predominant microbes enriched in the OVXH group (Supplementary Fig. 2b). Furthermore, random forest analysis highlighted key differentially abundant taxa, including *Roseburia*, *Oscillibacter*, *Allobaculum*, *Odoribacter*, *Muribaculum*, *Alistipes*, and *Lachnospiraceae_NK4A136* (Fig. 3b). Importantly, none of these differentially abundant genera are recognized periodontal pathogens, suggesting that the periodontitis-associated salivary microbiota may exert systemic effects indirectly via gut-mediated pathways rather than through direct translocation of oral pathobionts. Kyoto Encyclopedia of Genes and Genomes (KEGG) enrichment analysis revealed significant differences between the OVXH and OVXP groups, particularly in global and overview maps, carbohydrate metabolism, and amino acid metabolism pathways (Supplementary Fig. 2c). Notably, the periodontitis-associated salivary microbiota was associated with significant suppression of aromatic amino acid metabolism pathways (Supplementary Fig. 2d).

To determine whether alterations in the gut microbiota driven by the periodontitis-associated salivary microbiota contribute to the bone loss in OVX mice, we performed FMT. Recipient mice underwent gut microbiota depletion via a 2-week antibiotic regimen. Fecal microbiota from donor mice (OVXH and OVXP groups) were subsequently recurrently transplanted into recipient mice, which were designated the t-OVXH and t-OVXP groups, respectively (Fig. 3c). PCoA and hierarchical clustering of the recipient gut microbiota revealed distinct clustering patterns between the t-OVXH and t-OVXP groups (Fig. 3d, Supplementary Fig. 2e). Compared with t-OVXH recipients, t-OVXP recipients presented decreased relative abundances of *Roseburia* and *Oscillibacter* and increased abundances of *Lachnospiraceae_NK4A136* and *Allobaculum* (Supplementary Fig. 2f). These alterations closely mirrored the donor microbiota profiles. Notably, *Roseburia* exhibited the greatest feature importance in both the donor and the recipient cohorts (Supplementary Fig. 2g, h). Collectively, these findings validate the successful establishment of the recipient model.

**Fig. 4:**
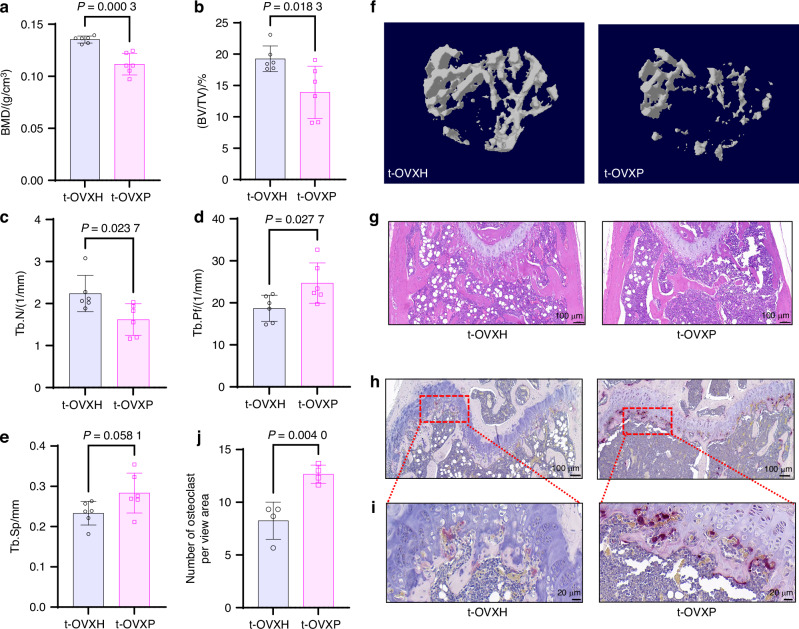
Gut dysbiosis induced by periodontitis-associated salivary microbiota exacerbates bone loss in OVX mice. Trabecular bone microarchitecture parameters of recipient mouse tibias: **a** bone mineral density (BMD), **b** bone volume fraction (BV/TV), **c** trabecular number (Tb.N), **d** trabecular pattern factor (Tb.Pf), and **e** trabecular separation (Tb.Sp). n = 6 per group. **f** Representative 3D reconstruction of tibial trabecular bone (basis for the calculations in **a**–**e**). **g**, **h** Representative H&E and TRAP staining of distal femoral sections (scale bar: 100 μm). **i** A magnified view of the region boxed in (**h**) (scale bar: 20 μm). **j** Quantification of TRAP-positive osteoclasts (n = 4 per group). The data are presented as the mean ± SD. *P* values were determined by Student’s t-test. All the statistical tests were two-sided

Compared with the t-OVXH group, the t-OVXP group displayed an increased severity of bone destruction, characterized by trabecular fragmentation and significantly reduced BMD (Fig. 4a–f). Quantitative analysis of trabecular microarchitecture revealed decreased BV/TV and Tb.N and increased Tb.Pf in the t-OVXP group (Fig. 4b–d). Histological analysis confirmed that the trabeculae were sparse and narrow (Fig. 4g), and TRAP staining revealed abundant osteoclasts in the growth plate region in the t-OVXP group (Fig. 4h–j). These data demonstrate that the gut microbiota mediates the exacerbation of bone loss triggered by the periodontitis-associated salivary microbiota in OVX mice.

### Impairment of tryptophan metabolism by periodontitis-associated salivary microbiota in OVX mice

**Fig. 5:**
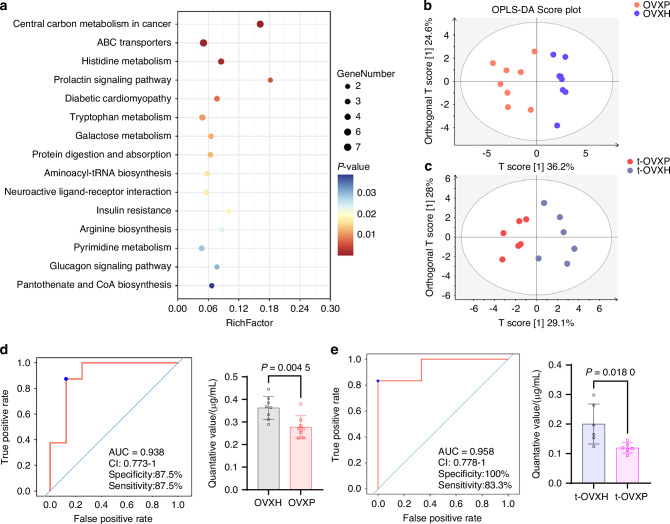
Periodontitis-associated salivary microbiota reduces the levels of tryptophan metabolites, including indole-3-lactic acid (ILA). **a** KEGG pathway enrichment analysis (bubble plot) of metabolomic data from cecal contents (OVXH vs. OVXP groups; n = 8 per group). OPLS-DA clustering of serum tryptophan metabolism profiles from **b** donor mice (OVXH vs. OVXP; n = 8 per group) and **c** recipient mice (t-OVXH vs. t-OVXP; n = 6 per group). **d**, **e** Receiver operating characteristic (ROC) analysis and serum ILA concentrations of the mice represented in (**b**, **c**). The data are presented as the mean ± SD. *P* values were determined by Student’s t-test. All the statistical tests were two-sided

Gut metabolites are intricately linked to host metabolic homeostasis. Metabolomic analysis of the cecal contents demonstrated significant differences between the OVXH and OVXP groups (Supplementary Fig. 3a). Volcano plot analysis identified 303 differentially abundant metabolites (VIP > 1, *P* < 0.05), comprising 172 downregulated (log_2_|FC| < 0) and 131 upregulated (log_2_|FC| > 0) metabolites in the OVXP group compared with the OVXH group (Supplementary Fig. 3b). KEGG pathway enrichment analysis of these differentially abundant metabolites revealed a marked enrichment of tryptophan metabolism (Fig. 5a). We subsequently conducted targeted metabolomics analysis on serum tryptophan metabolism in both recipient and donor mice. OPLS-DA revealed significant differences in tryptophan metabolism profiles between the OVXH and OVXP groups and between the t-OVXH and t-OVXP groups (Fig. 5b, c). Heatmap analysis revealed significantly reduced serum levels of tryptophan and related metabolites in both the OVXP and t-OVXP groups (Supplementary Fig. 3c, d). Receiver operating characteristic (ROC) curve were plotted, and the area under the curve (AUC) was used to evaluate the sensitivity and specificity in predicting event occurrence. Notably, the AUC values of ILA exceeded 0.9 in both recipient and donor mice, indicating high sensitivity and specificity (Fig. 5d, e). Furthermore, serum ILA levels did not significantly differ between the OVX and Sham groups (Supplementary Fig. 3e). These findings indicate that inhibition of tryptophan metabolism and a reduction in the abundance of its metabolite, ILA, are closely related to the exacerbation of osteoporosis by the salivary microbiota in periodontitis.

### Tryptophan metabolite ILA can decrease osteoclast differentiation in vitro

**Fig. 6:**
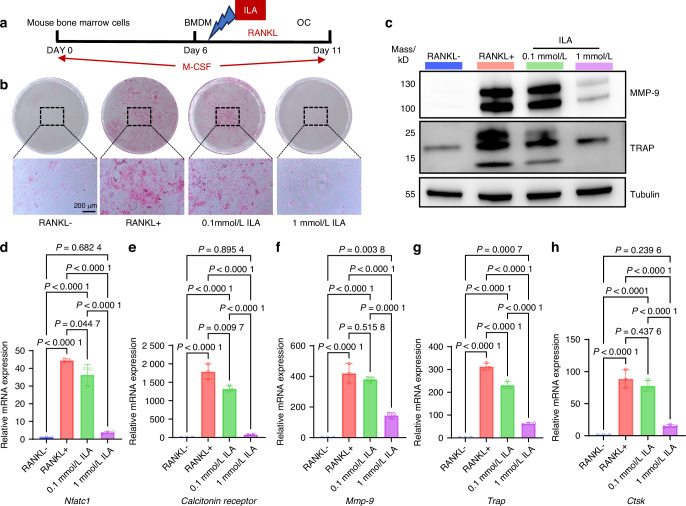
Effects of ILA on osteoclasts under RANKL-induced conditions in vitro. **a** Schematic diagram of the in vitro experimental design. **b** Representative images of TRAP-positive osteoclasts (scale bar: 200 μm). **c** Western blot analysis of MMP-9 and TRAP protein expression. **d**–**h** qPCR analysis of osteoclast-specific gene expression. The data are presented as the mean ± SD (n = 3 independent experiments). *P* values were determined by one-way ANOVA with Tukey’s test

We hypothesized that the decreased levels of ILA might be a key metabolite contributing to the exacerbation of bone destruction in OVX mice by the periodontitis-associated salivary microbiota. To validate this hypothesis, we began by isolating bone- marrow-derived macrophages (BMDMs) from C57BL/6 mice, identifying these cells by the expression of the macrophage surface markers F4/80 and CD11b. The percentage of cells positive for F4/80 and CD11b was greater than 90% (Supplementary Fig. 4a, b), confirming the successful induction of BMDMs. The cell viability of BMDMs treated with varying concentrations of ILA was then assessed using CCK-8 assays. Compared with the control treatment, low concentrations of ILA (1, 10, and 100 μmol/L) had no significant effect on BMDM proliferation or viability, whereas a high concentration (1 mmol/L) promoted BMDM proliferation (Supplementary Fig. 4c). These results indicate the good biocompatibility of ILA. To examine the effect of ILA on osteoclast differentiation and function, we provided ILA supplementation during osteoclast differentiation in an ex vivo culture system (Fig. 6a). TRAP staining and Western blot (WB) analysis demonstrated that ILA dose-dependently suppressed osteoclast formation and function (Fig. 6b, c), as further evidenced by the reduced expression of key markers associated with osteoclast activity and differentiation, including *Nfatc1*, *calcitonin receptor*, *Mmp-9*, *Trap* and *Ctsk* (Fig. 6d–h).

### ILA supplementation mitigates bone destruction induced by periodontitis-associated salivary microbiota in OVX mice

To further assess the in vivo impact of ILA, both ILA and salivary microbiota were administered to OVX mice via gavage (Supplementary Fig. 5a). At 9 weeks post-OVX surgery, micro-CT analysis revealed trabecular bone fragmentation, reduced BMD, and a sparse distribution of trabeculae in long bones, confirming the successful establishment of the osteoporosis model (Supplementary Fig. 5b). Although no significant difference in the alpha diversity of the gut microbiota was observed between the OVXP and OVXP-ILA groups, ILA supplementation tended to increase the Simpson index compared to that of the OVXH group (Supplementary Fig. 5c). Analysis of beta diversity revealed distinct clustering of gut microbiota communities among the OVXH, OVXP, and OVXP-ILA groups (Supplementary Fig. 5d). Random forest analysis revealed that ILA intervention significantly increased the relative abundance of the genera *Alistipes* and *Lactobacillus* (Supplementary Fig. 5e, f). Notably, *Alistipes* and *Lactobacillus* are recognized as tryptophan-metabolizing genera, with the latter performing critical probiotic functions.^23,24^

**Fig. 7:**
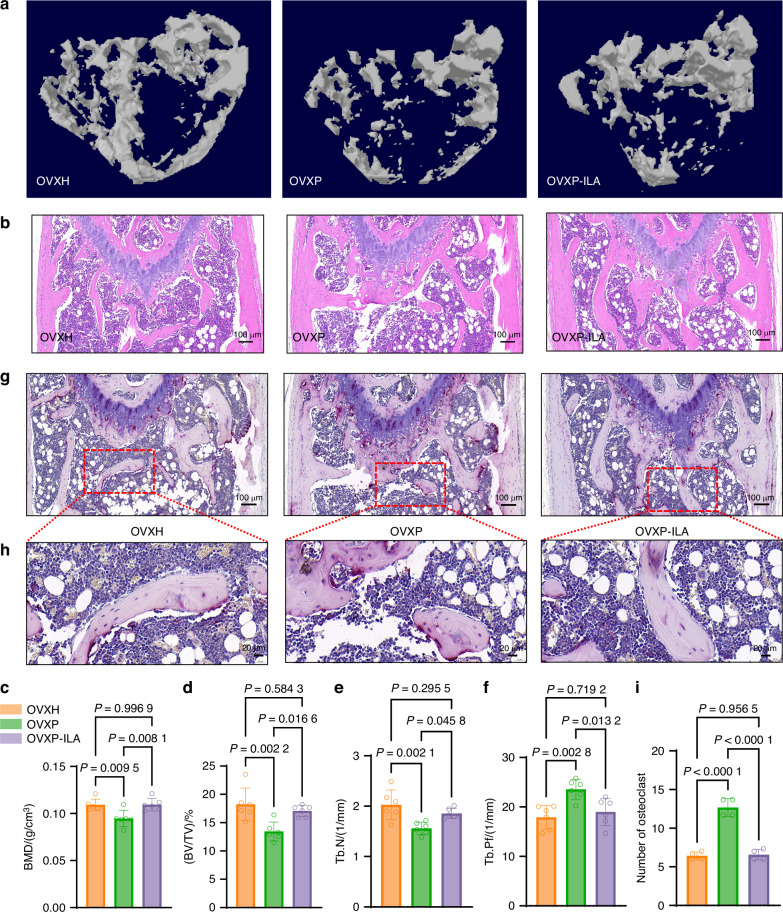
ILA supplementation alleviates bone loss induced by periodontitis-associated salivary microbiota in OVX mice. Representative micro-CT images (**a**) and H&E staining (**b**) of trabecular bone from OVX mice without (OVXH and OVXP groups) or with ILA (OVXP-ILA group) ILA treatment. Trabecular bone microarchitecture parameters: **c** bone mineral density (BMD), **d** bone volume fraction (BV/TV), **e** trabecular number (Tb.N), and **f** trabecular pattern factor (Tb.Pf) in the mice from (**a**) (*n* = 6 per group). **g** Representative TRAP staining of distal femoral sections (scale bar: 100 μm). **h** A magnified view of the region boxed in (**g**) (scale bar: 20 μm). **i** Quantification of TRAP-positive osteoclasts (*n* = 4 per group). The data are presented as the mean ± SD. *P* values were determined by one-way ANOVA with a Tukey’s test

Micro-CT and H&E staining demonstrated pronounced differences in bone microstructure. The OVXP group exhibited severe trabecular deterioration, characterized by reduced bone density, fragmentation, and a rod-like morphology. In contrast, compared with the OVXP controls, the OVXP-ILA group showed significant restoration of trabecular density and a plate-like trabecular structure (Fig. 7a, b). Quantitative analysis confirmed that ILA supplementation increased BV/TV by 1.3-fold and Tb.N by 1.2-fold while reducing Tb.Pf by 20% (Fig. 7c–f). Critically, no significant differences in BMD or trabecular parameters were observed between the OVXP-ILA and OVXH groups, indicating complete reversal of the osteopenia that had been induced by periodontitis-associated salivary microbiota. TRAP staining revealed a 52% reduction in osteoclast numbers following ILA intervention (Fig. 7g–i). Collectively, these findings demonstrate that ILA administration mitigates bone destruction induced by periodontitis-associated salivary microbiota in OVX mice by suppressing osteoclastogenesis.

## Discussion

Periodontopathic bacteria can influence systemic diseases by modulating the gut microbiota.^25–28^ In addition to the effects of individual pathogens, microbial communities exhibit synergistic interactions that enhance colonization, persistence, and pathogenicity.^29^ Therefore, to explore its impact on osteoporosis, we investigated the periodontitis-associated oral microbiota as an integrated entity. We found that patients with periodontitis exhibited significantly increased salivary microbiota diversity and elevated pathogenic bacterial abundance. Furthermore, we demonstrated that the exacerbation of bone resorption in OVX mice induced by the periodontitis-associated salivary microbiota was associated with gut microbiota dysbiosis. Critically, our findings indicate that the tryptophan metabolite ILA inhibits osteoclastogenesis, thereby mitigating the detrimental effects of periodontitis-associated salivary microbiota on bones in OVX mice.

The consensus hypothesis posits that a compromised epithelium in periodontal pockets serves as a pathological conduit, enabling bacterial translocation, LPS dissemination, and inflammatory mediator infiltration into the circulatory system, initiating a cascade that culminates in persistently elevated systemic inflammation; this pathway now represents the prevailing paradigm explaining the etiological link between periodontitis and systemic diseases.^6,30^ Previous studies in the ligature-induced periodontitis model have demonstrated that, in addition to bacteria directly translocating to distant tissues and exerting effects, inflammatory factors and immune cells activated by periodontitis can also enter the bloodstream and the gut, where they may act synergistically with bacteria to influence systemic diseases.^20,21^ To eliminate the confounding effects of these factors and specifically investigate the role of the oral–gut axis in the effect of periodontitis-associated salivary microbiota on OVX mice, we generated a model system by oral gavage of salivary microbiota.

Our study demonstrated that the salivary microbiota diversity in the periodontitis group was significantly greater than that in the healthy group, and the average age of the periodontitis group was also significantly greater. Multiple studies have suggested that the oral microbiota undergoes stage-specific changes throughout life.^31–33^ Some studies have indicated that the diversity index of the oral microbiota gradually increases during childhood, slightly fluctuates in young adulthood, and markedly decreases in old age.^34–37^ In contrast, other studies have reported opposing findings, showing that the composition of the oral microbiota is more diverse in elderly individuals.^38,39^ These conflicting results underscore the complexity of the oral microbiome. In the future, a comprehensive consideration of factors such as age and oral health status is needed to further explore the patterns of oral microbiome variation. In the present study, significant differences in salivary microbiota diversity and composition were observed between periodontitis patients and periodontally healthy individuals. However, following oral administration of salivary microbiota, we detected no significant increases in periodontitis pathogens within the gut microbiota of OVX mice. Critically, validation using an FMT model confirmed the essential role of the gut microbiota in this process. Thus, we propose that the periodontitis-associated salivary microbiota aggravates bone destruction in OVX mice by inducing gut dysbiosis.

Accumulating evidence highlights the critical role of the gut microbiota as a mediator of bone metabolism. The gut microbiota regulates skeletal homeostasis by modulating osteoclast and osteoblast activity through its microbial communities and their metabolites.^17,40,41^ Notably, the abundances of bacterial genera associated with beneficial functions—including *Rothia*, *Oscillibacter*, *Odoribacter*, and *Alistipes* (which have been implicated in amino acid metabolism, short-chain fatty acid production, and systemic anti-inflammatory effects)—were significantly reduced in OVXP mice.^42–45^ Conversely, *Allobaculum*, a genus linked to exacerbated colonic inflammation,^46^ was significantly enriched in this group. These shifts indicate that gavage with periodontitis-associated salivary microbiota induces gut dysbiosis with detrimental consequences.

A recent study has revealed significantly increased abundances of multiple pathogenic bacteria in both the saliva and feces of periodontitis patients. After periodontal treatment, the abundance of these pathogens can be markedly reduced, suggesting that periodontal therapy plays a significant regulatory role in modulating oral and gut microbiota.^47^ However, even after comprehensive treatment, the levels of *Porphyromonas*, *Tannerella*, and *Treponema* remain higher in the saliva of periodontitis patients than in that of healthy controls.^47^ The persistent proliferation of these pathogens in periodontal pockets may drive recurrent dysbiosis in both the oral and gut ecosystems.^48^ In addition, systemic antibiotic therapy combined with periodontal treatment has been shown to achieve better clinical outcomes than periodontal treatment alone.^49^ However, a recent study has indicated that systemic antibiotic use can lead to intestinal injury and exacerbate alveolar bone loss. Even after the discontinuation of antibiotics, the gut microbiota in periodontitis-afflicted mice failed to return from dysbiotic to normal, while the pathogenicity of the oral microbiota increased.^50^ Therefore, targeting the gut microbiota and its metabolites represents a promising novel approach to effectively mitigate systemic adverse effects of periodontitis. Bacteria-derived metabolites critically modulate host‒microbial interactions and the development of metabolic diseases.^51^ Wikoff et al. demonstrated that the gut microbiota influences approximately 10% of serum metabolites, with particularly pronounced effects on amino acid metabolism.^52^ Among these, tryptophan and the indole derivatives produced from it by the gut microbiota play physiologically significant roles in regulating bone homeostasis.^53–55^ In our study, tryptophan metabolism was significantly inhibited following the administration of periodontitis-associated salivary microbiota by gavage. A larger area under the ROC curve corresponds to greater diagnostic accuracy, and when this value exceeds 0.9, it indicates strong diagnostic utility. Notably, the serum ILA concentration was markedly reduced in both OVXP and t-OVXP mice and exhibited high sensitivity and specificity. These findings suggest that ILA functions as a pivotal effector metabolite, mediating the effects of periodontitis on distal organs via the oral–gut axis.

Under physiological conditions, bone resorption and formation are in dynamic equilibrium. This study demonstrated that gavage with periodontitis-associated salivary microbiota significantly increased the number of osteoclasts in murine bone. Osteoclasts—specialized multinucleated myeloid cells that differentiate from monocyte/macrophage precursors adjacent to bone surfaces—are the principal mediators of bone resorption.^56^ Our findings revealed that ILA supplementation markedly inhibited osteoclast differentiation and function both in vitro and in vivo. Consequently, ILA improved BMD and preserved trabecular bone structural integrity in OVX mice treated with periodontitis-associated salivary microbiota. Critically, the role of ILA in bone metabolism remains unreported. This study provides the first elucidation of the critical function of ILA in bone metabolism, offering novel insights for exploring its role in skeletal homeostasis.

This study has several limitations. First, clinical analysis was constrained by the use of a single batch of saliva samples for both 16S rRNA sequencing and subsequent gavage experiments, limiting the sample size. Future studies should incorporate larger cohorts. Second, while ILA directly inhibits osteoclastogenesis, it may also indirectly affect bone via immune modulation. ILA has been demonstrated to alleviate intestinal inflammation and maintain gut homeostasis.^57–59^ Given the close interactions between the immune and skeletal systems, this dual pathway warrants exploration.^60,61^ Additionally, as an aryl hydrocarbon receptor (AhR) ligand,^57^ ILA might signal through AhR in osteoclast precursors, a possibility that needs to be investigated in the future. Finally, 16S sequencing may fail to capture subtle compositional differences and the roles of specific bacterial species. Metagenomic sequencing should be employed in future work to address these limitations.

In conclusion, this study demonstrated that periodontitis patients exhibit significantly increased diversity and pathogen abundance within their salivary microbiota. Critically, periodontitis-associated salivary microbiota aggravated bone loss in OVX mice by inducing gut microbiota dysbiosis. This effect was mediated, at least in part, by depletion of the osteoprotective metabolite ILA, thereby promoting osteoclast formation. ILA supplementation effectively counteracted this bone deterioration by inhibiting osteoclastogenesis and mitigating the detrimental skeletal effects of the periodontitis-associated salivary microbiota in OVX mice. Collectively, these findings highlight the modulation of the gut microbiota and its metabolites as promising therapeutic targets to mitigate the adverse effects of periodontitis on systemic bone metabolism.

## Materials and methods

### Clinical participants

A cohort of 38 participants was recruited from Nanjing Stomatological Hospital, Affiliated Hospital of Medical School, Nanjing University. The inclusion criteria for healthy donors (H group) were as follows: (1) age ≥18 years; (2) no history of tooth loss due to periodontitis; and (3) no clinical attachment loss. The inclusion criteria for patients with periodontitis (P group) were as follows: (1) age ≥18 years; (2) presence of ≥20 remaining teeth; and (3) a diagnosis of stage III/IV periodontitis affecting ≥ 30% of the teeth. The exclusion criteria (applied to all participants) were as follows: (1) periodontal treatment within the preceding 6 months; (2) the use of antibiotic, nonsteroidal drugs or therapeutic mouthwashes within the preceding 6 months; (3) pregnancy; and (4) a history of systemic diseases (e.g., gastrointestinal disorders, diabetes, or rheumatoid arthritis). The study protocol was approved by the Ethics Committee of Nanjing Stomatological Hospital, Affiliated Hospital of Medical School, Nanjing University (Approval No: NJSH-2023NL-081). All participants provided written informed consent for the collection and use of their saliva samples and clinical data.

### Collection and processing of saliva samples

Unstimulated saliva samples were collected from all participants as previously described.^62^ Briefly, participants were instructed to gently spit saliva into sterile 50 mL tubes every 1–2 min to collect unstimulated saliva samples. A 1 mL aliquot of each sample was transferred to cryovials, flash-frozen in liquid nitrogen, and stored at −80 °C for subsequent analysis. The remaining saliva was centrifuged at 1 000 r/min for 10 min. The supernatant was then mixed with an equal volume of phosphate-buffered saline (PBS) containing 20% glycerol (v/v) and stored at −80 °C. Prior to oral administration, the samples were thawed rapidly in a 37 °C water bath. Saliva from individuals within the same group was pooled and centrifuged at 3 300 × *g* for 10 min, after which the resulting pellet was resuspended in PBS for use in animal experiments.

### Animals

The animal protocols were approved by the Animal Welfare and Ethics Committee of Nanjing Agriculture University (Approval No. PZW2024028). Seven-week-old female C57BL/6 mice were obtained from Vital River Laboratories (Beijing) and housed under specific-pathogen-free (SPF) conditions at the university facility. The animals were maintained at a constant temperature (22 °C ± 1 °C and 55% ± 5% relative humidity on a 12/12-h light/dark cycle.

#### OVX mouse model

After a 1-week acclimation period, the mice underwent bilateral ovariectomy under isoflurane inhalation anesthesia. Sham-operated control mice (the Sham group) underwent anesthesia and peri-ovarian adipose tissue removal. All the mice were euthanized for analyses at 9 weeks after surgery.

#### Salivary microbiota gavage model

Sixteen OVX mice were randomly assigned to two groups (*n* = 8 per group): OVXH and OVXP. Mice received 200 μL of resuspended salivary microbiota via oral gavage every other day for 7 weeks. The OVXH group received microbiota from healthy donors, while the OVXP group received microbiota from periodontitis patients. Beginning in the 4th week of gavage, fresh fecal pellets were collected every 2 days for subsequent FMT. The mice were euthanized at the end of experimental for analysis.

#### Antibiotic-treated mice and FMT model

Two weeks post-ovariectomy, twelve mice received an antibiotic cocktail (1 g/L ampicillin sodium, 1 g/L metronidazole, 1 g/L neomycin sulfate, 0.5 g/L vancomycin) in their drinking water for 2 weeks to deplete the gut microbiota.^63^ The mice were then randomly divided into the t-OVXH and t-OVXP groups (n = 6 per group) and received FMT every other day for 3 weeks. For FMT preparation, fecal samples from the corresponding donor groups (OVXH or OVXP) were pooled (six pellets per batch), homogenized in PBS by vortexing, and centrifuged (1 000 r/min, 5 min). The supernatant was immediately administered via oral gavage to recipient mice. All the mice were euthanized at the end of experimental for analysis.

#### ILA gavage model

Two weeks post-ovariectomy, eighteen mice were randomly divided into three groups (n = 6 per group): OVXH, OVXP, and OVXP-ILA. Concurrent with the salivary microbiota gavage regimen, mice received either ILA (20 mg/kg body weight) or vehicle (PBS) by oral gavage every other day for 7 weeks until euthanasia.

### Micro-CT analysis

Mouse tibiae were fixed in 4% paraformaldehyde for 48 h and subsequently scanned using a Skyscan 1176 micro-computed tomography system (Bruker, Germany). Scans were acquired at a spatial resolution of 18 µm with the X-ray source operating at 50 kV and 455 µA. The acquired scans were aligned using Data Viewer software to ensure uniform orientation across all the samples. For trabecular bone analysis, a volume of interest (VOI) encompassing 30 consecutive slices was selected, starting 20 slices distal to the growth plate. Three-dimensional reconstructions were generated, and trabecular bone parameters—including BV/TV, Tb.N, Tb.Sp, Tb.Th and Tb.Pf—were quantified using CTAn software. Three-dimensional visualization was performed using CTvox software. BMD was calibrated using hydroxyapatite phantoms with known BMD values (0.25 g/cm³ and 0.75 g/cm³) as reference standards.

### Bone histomorphometric analysis

Following 48 h fixation in 4% paraformaldehyde, femurs were decalcified in EDTA and embedded in paraffin blocks. Longitudinal sections (4 μm thick) were prepared and stained with H&E or TRAP. Images were digitized using a PANNORAMIC MIDI scanner (3DHISTECH, Hungary) and visualized with Caseviewer software. For TRAP-stained sections, four randomly selected fields (80× magnification) per sample were captured. The number of TRAP-positive multinucleated osteoclasts per field was quantified, and the mean count was calculated.

### 16S rRNA gene amplicon sequencing

Human saliva and mouse cecal contents were collected, flash-frozen in liquid nitrogen, and stored at −80 °C. Bacterial DNA was extracted using the MagPure Soil DNA Extraction Kit (Magen, China). The V3-V4 region of the 16S rRNA gene was amplified with the following primers: forward primer 5′-TACGGRAGGCAGCAG-3′ and reverse primer 5′-AGGGTATCTAATCCT-3′. Amplicons were electrophoresed to verify specificity, and samples that passed this quality check were quantified using a Qubit dsDNA Assay Kit (Thermo Fisher Scientific, USA). Sequencing was conducted on the Illumina NovaSeq 6000 platform, generating 250 bp paired-end reads. Library preparation, sequencing, and data processing were conducted by OE Biotech Co., Ltd. (Shanghai, China). Within the QIIME2 bioinformatics platform, representative reads of amplicon sequence variants (ASVs) were selected and taxonomically annotated by alignment against the SILVA database. Microbial diversity and community structure were assessed using alpha diversity indices (Chao1, Shannon, Simpson, and observed species) and PCoA, respectively. Statistically significant biomarkers driving intergroup microbial disparities were identified via LEfSe by applying a linear discriminant analysis (LDA) score threshold >2.0 and a *P* value < 0.05. Key bacterial genera capable of distinguishing between groups were identified by evaluating the predictive contribution of the 30 taxa with the highest relative abundance through random forest analysis.

### Untargeted metabolomics

Untargeted metabolomic analysis of the cecal contents was performed using liquid chromatography‒mass spectrometry (LC‒MS) as previously described.^64^ Briefly, 60 mg of each sample was transferred to a 1.5 mL Eppendorf tube. Afterward, 600 μL of methanol‒water solution (4:1, v/v) containing 4 μg/mL L-2-chlorophenylalanine as an internal standard was added. The mixture was homogenized using a tissue grinder, incubated in an ice-water bath for 10 min, and centrifuged at 13 000 r/min for 10 min at 4 °C. The supernatant was collected, transferred to an LC‒MS vial, and evaporated to dryness under nitrogen gas. The residue was reconstituted in methanol‒water solution (1:4, v/v), filtered through a 0.22 μm membrane filter, and subjected to chromatographic separation using an ACQUITY UPLC HSS T3 column (2.1 mm × 100 mm, 1.8 μm). The mobile phases consisted of (A) 0.1% formic acid in water and (B) 0.1% formic acid in acetonitrile. Mass spectrometric detection was performed in both positive and negative ion modes.

The raw data were processed using Progenesis QI software (version 2.3). Metabolites were identified by matching the data to online databases, including the Human Metabolome Database (HMDB), METLIN, and LIPIDMAPS (Version 2.3). Orthogonal partial least squares discriminant analysis (OPLS-DA) was implemented to discriminate metabolic profiles between groups. KEGG pathway analysis (https://www.kegg.jp) was subsequently conducted to explore metabolic pathways enriched in differentially expressed metabolites.

### Targeted quantification of tryptophan metabolites

Serum tryptophan metabolites were quantified using LC‒MS. To this end, a 100 μL aliquot of serum was mixed with 100 μL of 80% methanol. The mixture was vortexed and then supplemented with 900 μL of 10% methanol. After centrifugation at 12 000 r/min and 4 °C for 5 min, 100 μL of the supernatant was mixed with 100 μL of a 20 ppb Trp-d5 solution (isotope-labeled internal standard) and vortexed for 30 s. Subsequently, 150 μL of this mixture was transferred to an analytical vial for LC‒MS detection.

Chromatographic separation was achieved using an ACQUITY UPLC HSS T3 column (2.1mm × 150 mm, 1.8 μm; Waters, USA). The separated compounds were introduced into a mass spectrometer equipped with an electrospray ionization (ESI) source operating in positive ion mode. The ESI source parameters were set as follows: ion source temperature = 450 °C, ion source voltage = 4 500 V, collision gas = 10 psi, curtain gas = 30 psi, atomizing gas = 50 psi, and auxiliary gas = 50 psi. Mass spectrometric detection was performed using multiple reaction monitoring (MRM).

### Primary cell culture

BMDMs were isolated and cultured as previously described.^65^ Briefly, bilateral femurs and tibiae were dissected from 6-week-old female mice, and bone marrow cells were flushed from the medullary cavities. The cell suspension was filtered through a 70 μm strainer and cultured in complete α-MEM supplemented with 10% FBS (Gibco, USA), 1% penicillin‒streptomycin antibiotic cocktail (Gibco, USA), and 25 ng/mL recombinant murine macrophage colony-stimulating factor (M-CSF) (Peprotech, USA). On Day 6, differentiation was induced by adding 50 ng/mL receptor activator of nuclear factor-κB ligand (RANKL) (Peprotech, USA) and ILA (0, 0.1, or 1 mmol/L) (MCE, USA) to the culture medium. After 5 days of incubation, the cells were subjected to TRAP staining, RNA extraction, and protein isolation for subsequent analyses.

### Flow cytometry assay

BMDMs were harvested from culture dishes and transferred to flow cytometry tubes. After centrifugation (300 × *g*, 5 min), the supernatant was discarded. Cells were incubated with an anti-CD16/32 antibody for 5 min at room temperature to block Fc receptors, after which they were stained with FITC-conjugated anti-F4/80 and PE-conjugated anti-CD11b antibodies. After two washes with PBS and resuspension, the samples were analyzed using a flow cytometer. The data were processed and analyzed with FlowJo software (10.9.0). BMDMs were identified by their positivity for F4/80 and CD11b. All antibodies were purchased from eBioscience.

### TRAP staining of cells

Cells were fixed with 4% paraformaldehyde for 30 min and rinsed with distilled water. Afterward, 0.2% Triton X-100 solution was added for cell permeabilization. The TRAP staining solution was freshly prepared according to the manufacturer’s instructions. The cells were incubated with the staining solution at 37 °C for 30 min and then rinsed twice with distilled water. Finally, images were captured using both a stereomicroscope (Nikon, Japan) and an inverted microscope (Nikon, Japan), and the number of TRAP-positive multinucleated cells per well was quantified.

### Cellular RNA extraction and RT-qPCR

Following 5 days of RANKL stimulation, total RNA was extracted using the SteadyPure Universal RNA Extraction Kit (Accurate Biology, China). cDNA was synthesized from 1 μg of RNA using the HiScript III RT SuperMix Kit for qPCR (Vazyme, China). RT-qPCR was then performed with ChamQ Universal SYBR qPCR Master Mix (Vazyme, China) on a ViiA 7 Real-Time PCR System (Thermo, USA). The primer sequences are provided in Supplementary Table S2. Target gene expression was quantified via the 2^−ΔΔCt^ method and normalized to the expression of β-actin.

### Western blot

Total cellular proteins were extracted using RIPA lysis buffer (Beyotime, China) supplemented with 1% protease inhibitor (Beyotime, China). Equal amounts of protein were separated by sodium dodecyl sulfate‒polyacrylamide gel electrophoresis (Beyotime, China) and transferred onto PVDF membranes (Millipore, USA) using a semi-dry transfer system. The membranes were blocked with 5% BSA in TBST for 1 h at room temperature and then incubated overnight at 4 °C with primary antibodies against TRAP (1:1 000; Proteintech, China) and MMP-9 (1:1 000; Abcam, USA). β-Tubulin (1:2 000; Abmart, China) served as the loading control. After being incubated with a horseradish peroxidase (HRP)-conjugated secondary antibody for 1 h, the membranes were washed three times with TBST. The protein bands were visualized using ECL detection reagent on a Tanon-5200 chemiluminescence imaging system (Tanon, China).

### Statistical analysis

The data are presented as the mean ± standard deviation (SD) unless otherwise indicated. If normality and homogeneity of variance were confirmed, pairwise differences between groups were analyzed using an independent-samples *t*-test; nonnormally distributed data were assessed using the Mann‒Whitney U test. For comparisons across more than two groups, one-way ANOVA was performed, followed by post hoc analysis using Dunnett’s t-test or the Tukey test. Statistical analyses were conducted in GraphPad Prism 9.0 (GraphPad, CA, USA), with *P* < 0.05 considered to indicate statistical significance.

## Supplementary information

Supplementary Information

## Data Availability

16S rRNA gene sequence data generated in this study have been deposited in and are available from the NCBI Sequence Read Archive under accession number PRJNA1285097.
