# Supplementary material for: Periodontitis-associated salivary microbiota exacerbates systemic osteoclastogenesis via gut modulation and tryptophan metabolism suppression in ovariectomized mice
Source: Int J Oral Sci. 2026 Jan 27;18:14. doi: 10.1038/s41368-025-00415-2 (PMC12834969; doi:10.1038/s41368-025-00415-2)
Supplement: Supplementary file 1 — Supplementary Information [file 41368_2025_415_MOESM1_ESM.pdf]

Supplementary Information for

Periodontitis-Associated Salivary Microbiota Exacerbates Systemic Osteoclastogenesis via Gut Modulation and Tryptophan Metabolism Suppression in Ovariectomized mice

Nannan Wang[1†], Jun Qian[1†], Min Wang[1], Lili Li[1], Wenzheng Liao[2], Rixin Chen[1], Hua Nie[1], Ruiyang Ge[1], Fangfang Sun[1*], Fuhua Yan[1*]

[1] Nanjing Stomatological Hospital, Affiliated Hospital of Medical School, Institute of Stomatology, Nanjing University, Nanjing, China

[2] Shenzhen Hospital, Southern Medical University, Shenzhen, China

[†] Nannan Wang and Jun Qian contributed equally to this work.

[*] Corresponding author: Fuhua Yan and Fangfang Sun.

Fuhua Yan, Nanjing Stomatological Hospital, Affiliated Hospital of Medical School, Institute of Stomatology, Nanjing University, 30 Zhongyang Road, Nanjing, Jiangsu 210008, China. Tel +86-25-83620253. E-mail address: yanfh@nju.edu.cn.

Fangfang Sun, Nanjing Stomatological Hospital, Affiliated Hospital of Medical School, Institute of Stomatology, Nanjing University, 30 Zhongyang Road, Nanjing, Jiangsu 210008, China. Tel +86-25-83620123. E-mail address: sff0517@163.com

SUPPLEMENTARY FIGURES

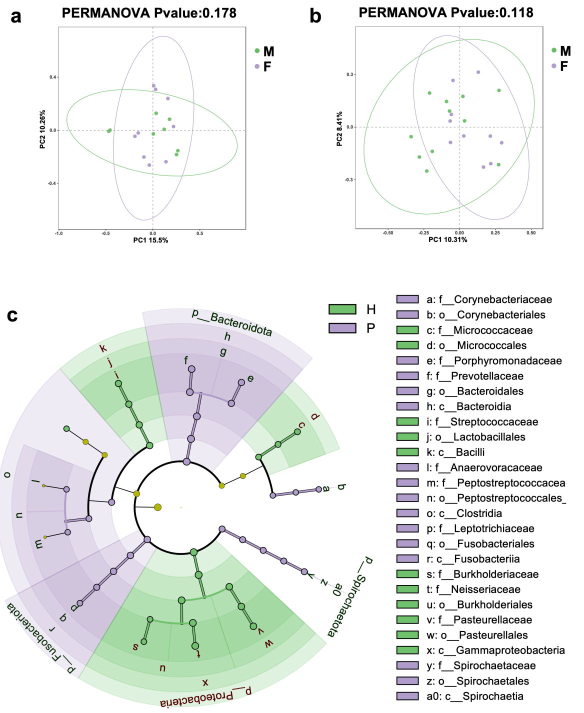

Supplementary Fig. 1 Microbial community analysis in health and periodontitis (a) Principal coordinate analysis (PCoA) of microbial communities in males (M group, n = 8) and females (F group, n = 9) among healthy donors, based on the binary Jaccard distance. (b) PCoA of microbial communities in males (M group, n = 11) and females (F group, n = 10) with periodontitis. (c) Linear discriminant analysis effect size (LEfSe) showing the discriminant bacterial features between groups H and P, based on LDA scores. *P* values were determined by PERMANOVA using the binary Jaccard distance (a and b) and LEfSe (c).

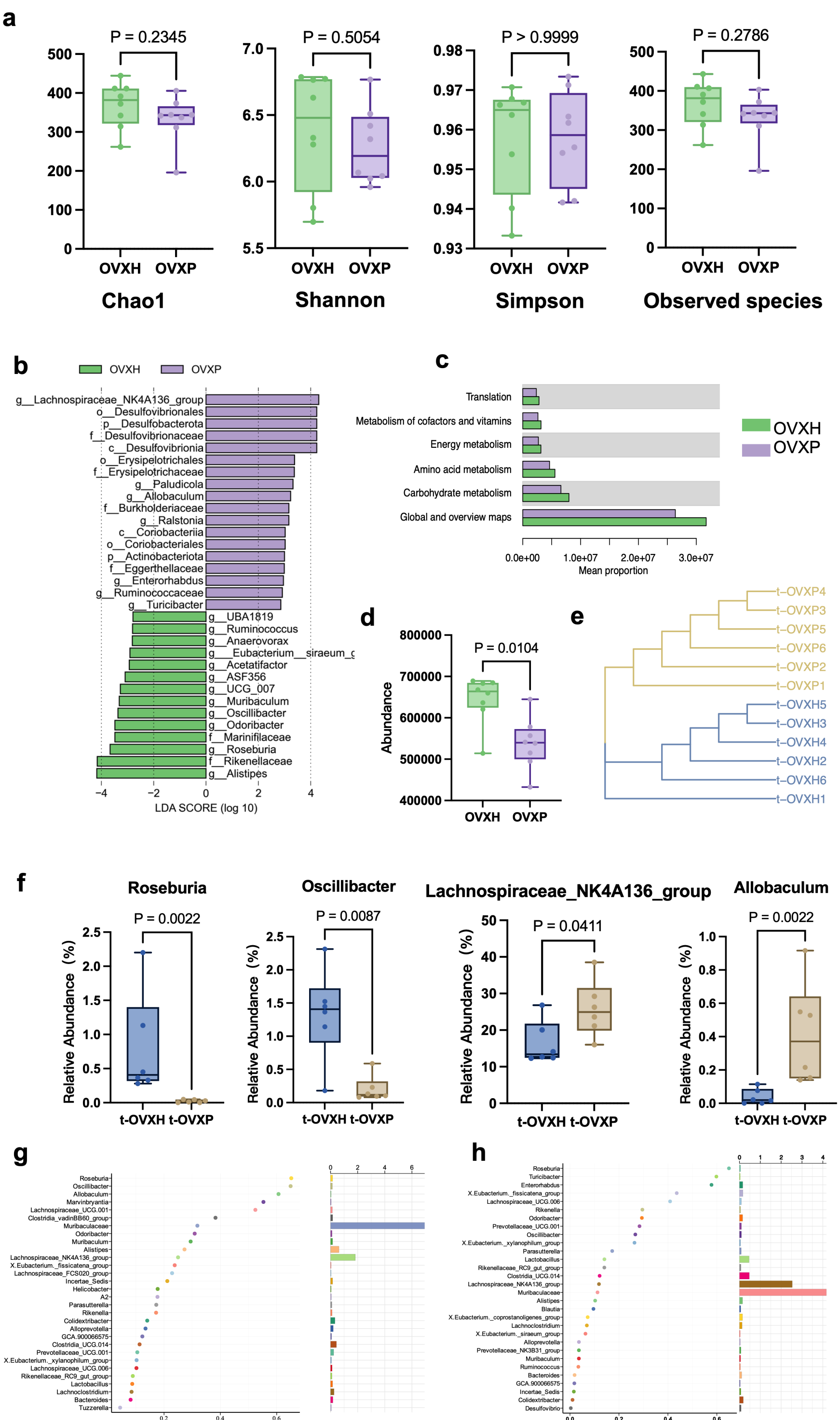

Supplementary Fig. 2 Oral administration of salivary microbiota alters gut microbiota composition

(a) Alpha diversity indices (Chao 1, Shannon, Simpson, observed species) of gut microbiota in OVX mice following oral administration of salivary microbiota from periodontitis patients (OVXP group, n = 8) or healthy donors (OVXH group, n = 8).

Box plot depict the median, interquartile range, minimum, and maximum values. (b) LEfSe (LDA effect size) cladograms illustrating differentially enriched bacterial taxa between the OVXH and OVXP groups. (c) Differentially abundant KEGG pathways between groups, with the average pathway abundance shown. (d) COG functional profiles for aromatic amino acid in OVXH vs. OVXP groups. Box plot depict the median, interquartile range, minimum, and maximum values. (e) Microbial beta diversity in recipient mice (t-OVXH and t-OVXP groups, n = 6 per group) assessed by hierarchical clustering (binary Jaccard distance). (f) Differentially abundant donor-derived genera in recipient mice. Box plots depict the median, interquartile range, minimum, and maximum. (g-h) The random forest analysis for the recipient (OVXH and OVXP) and donor (t-OVXH and t-OVXP) groups, respectively. *P* values were determined by Mann-Whitney test (a, d and f). All the statistical tests were two-sided.

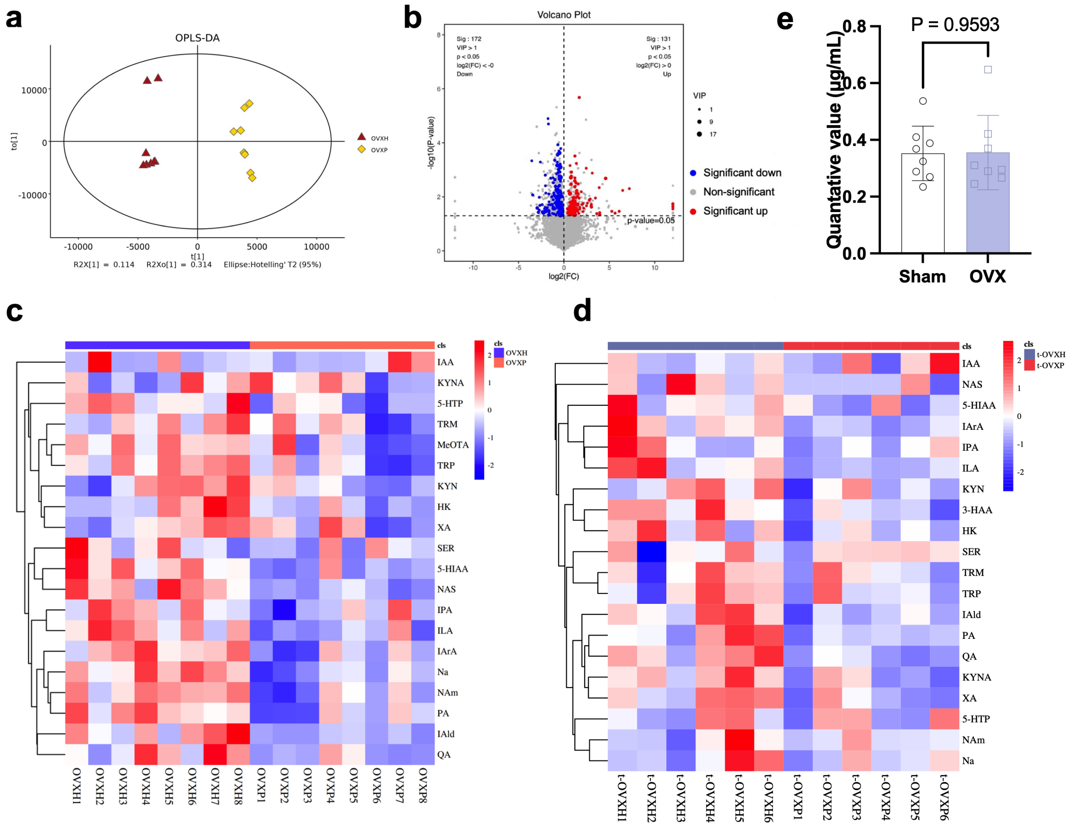

Supplementary Fig. 3 Metabolome analysis in OVX mice

(a) OPLS-DA score plot comparing cecal metabolomic profiles between OVXH and OVXP groups (n = 8 per group). (b) Volcano plot identifying significantly differential

metabolites in OVXH vs. OVXP groups. (c-d) Hierarchically clustered heatmaps of serum tryptophan-related metabolites corresponding to recipient (OVXH and OVXP) and donor (t-OVXH and t-OVXP) groups, respectively. (e) Effect of Ovariectomy on Serum ILA Concentration. *P* values were determined by Student's t-test.

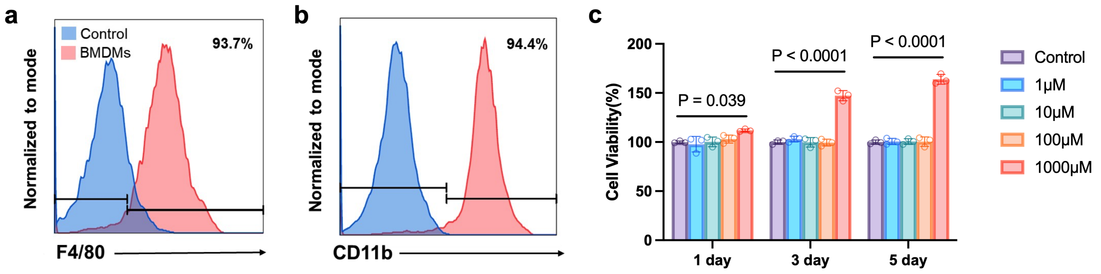

Supplementary Fig. 4 Characterization of BMDMs and analysis of ILA effects on cell viability

(a-b) Flow cytometry analysis of bone-marrow-derived macrophages (BMDMs). Blue: control cells; red: maker-positive cells). (c) Cell viability (CCK-8 assay) in BMDMs, measured on Days 1, 3, and 5. *P* values were determined by one-way ANOVA with Dunnett's t test.

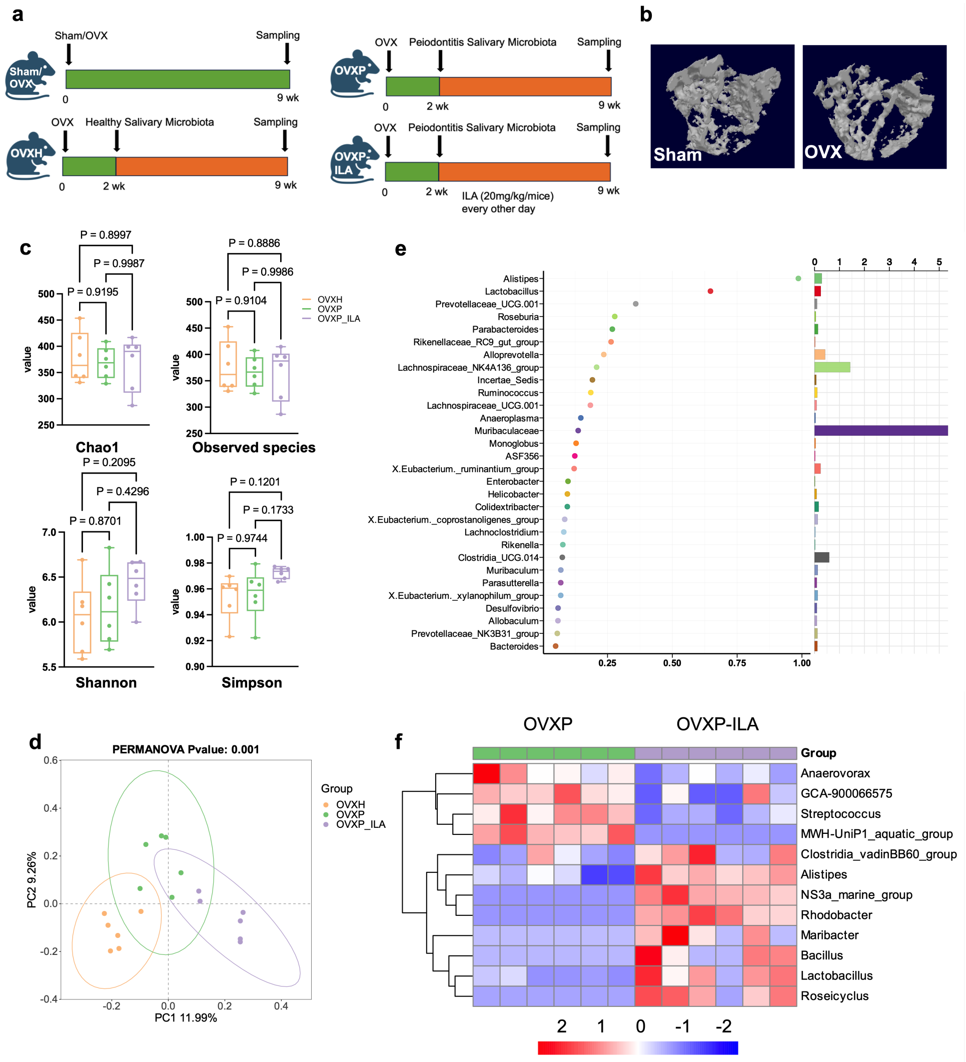

Supplementary Fig. 5 ILA supplementation alters gut microbiota composition

(a) Schematic of the experimental design. (b) Representative micro-CT images of tibial trabecular bone from Sham and OVX mice. (c) Alpha diversity indices (Chao 1, Shannon, Simpson and Observed species) of gut microbiota in OVX mice not treat (OVXH and OVXP groups) or treated with ILA (OVXP-ILA group). Box plot depict the median, interquartile range, minimum, and maximum values (n = 6 per group). (d) PCoA of microbial communities comparing OVXH, OVXP and OVXP-ILA groups, based on the binary Jaccard distance. (e) Discriminative bacteria identified by Random Forest Analysis. (f) Hierarchically clustered heatmap of significantly different genera. *P* values were determined by Mann-Whitney test (c) and PERMANOVA using the binary Jaccard distance (d). All statistical tests were two-sided.

Supplementary Table 1. Clinical characteristics of enrolled participants

**Characteristic**	**Healthy controls**	**Periodontitis**	**P value**
N (%)	17 (44.74%)	21 (55.26%)	
Age, years	30.35±4.37	43.95±10.38	<0.0001
Gender, n (%)			ns
Male	8（47.06%）	11（52.38%）	
Female	9（52.94%）	10（47.62%）	

P-Values are determined by Chi-Square test for Gender and Student's t test for Age.

Supplementary Table 2. Sequences of the primers used in this study

**Gene**	**Forward primer (5'-3')**	**Reverse primer (3'-5')**
Trap	CCAGCGACAAGAGGTTCC	AGAGACGTTGCCAAGGTGAT
NFATc1	CACACACCCCGCATGTCA	CGGGCCGCAAAGTTTCTC
Mmp-9	CTCTGCTGCCCCTTACCAG	CACAGCGTGGTGTTCGAATG
Ctsk	CCAGTGGGAGCTATGGAAGA	AAGTGGTTCATGGCCAGTTC
Calcitonin receptor	TGGTTGAGGTTGTGCCCA	CTCGTGGGTTTGCCTCATC
β-actin	GTGCTATGTTGCTCTAGACTTCG	ATGCCACAGGATTCCATACC
